# Selective killing of cancer cells harboring mutant RAS by concomitant inhibition of NADPH oxidase and glutathione biosynthesis

**DOI:** 10.1038/s41419-021-03473-6

**Published:** 2021-02-16

**Authors:** Muyun Liu, Dan Wang, Yongde Luo, Lianghao Hu, Yawei Bi, Juntao Ji, Haojie Huang, Guoqiang Wang, Liang Zhu, Jianjia Ma, Eunice Kim, Catherine K. Luo, James L. Abbruzzese, Xiaokun Li, Vincent W. Yang, Zhaoshen Li, Weiqin Lu

**Affiliations:** 1 Department of Gastroenterology, Changhai Hospital, Shanghai, China; 2 Department of Gastroenterology, No. 905 Hospital, Shanghai, China; 3 Division of Gastroenterology and Hepatology, Department of Medicine, Stony Brook University, Stony Brook, NY USA; 4 School of Pharmaceutical Sciences & The First Affiliated Hospital of Wenzhou Medical University, Wenzhou, Zhejiang China; 5 Division of Medical Oncology, Department of Medicine, Duke Cancer Institute, Duke University, Durham, NC USA

**Keywords:** Chemotherapy, Oncogenes

## Abstract

Oncogenic RAS is a critical driver for the initiation and progression of several types of cancers. However, effective therapeutic strategies by targeting RAS, in particular RAS^G12D^ and RAS^G12V^, and associated downstream pathways have been so far unsuccessful. Treatment of oncogenic RAS-ravaged cancer patients remains a currently unmet clinical need. Consistent with a major role in cancer metabolism, oncogenic RAS activation elevates both reactive oxygen species (ROS)-generating NADPH oxidase (NOX) activity and ROS-scavenging glutathione biosynthesis. At a certain threshold, the heightened oxidative stress and antioxidant capability achieve a higher level of redox balance, on which cancer cells depend to gain a selective advantage on survival and proliferation. However, this prominent metabolic feature may irrevocably render cancer cells vulnerable to concurrent inhibition of both NOX activity and glutathione biosynthesis, which may be exploited as a novel therapeutic strategy. In this report, we test this hypothesis by treating the HRAS^G12V^-transformed ovarian epithelial cells, mutant *KRAS*-harboring pancreatic and colon cancer cells of mouse and human origins, as well as cancer xenografts, with diphenyleneiodonium (DPI) and buthionine sulfoximine (BSO) combination, which inhibit NOX activity and glutathione biosynthesis, respectively. Our results demonstrate that concomitant targeting of NOX and glutathione biosynthesis induces a highly potent lethality to cancer cells harboring oncogenic RAS. Therefore, our studies provide a novel strategy against RAS-bearing cancers that warrants further mechanistic and translational investigation.

## Introduction

Abnormal activation of RAS signaling pathways by the acquisition of oncogenic mutations in *KRAS*, *HRAS*, or *NRAS* is frequently observed in human cancers^1–4^. Given the difficulty in developing a clinically effective RAS inhibitor as well as the complexity of RAS downstream pathways, a growing number of studies have been focusing on metabolic vulnerabilities conferred by oncogenic RAS^5–9^. Previous studies have shown that oncogenic RAS activation significantly augmented the activities of NOXs, which increase cellular ROS levels, leading to redox dysregulation and aberrant metabolic alterations^10–13^. NOXs are a group of seven membrane-bound multi-component enzymes, namely NOX1, NOX2 (gp91phox), NOX3, NOX4, NOX5, DUOX1, and DUOX2, capable of oxidizing NADPH to NADP^+^, leading to the generation of superoxide by one-electron reduction of oxygen^14^. The bioactive complexes of NOXs, e.g., NOX1 to NOX3, comprise the homologous cytosolic subunits, such as NOX organizer subunits (NOXO1 and p47phox), NOX activator subunits (NOXA1 and NOXA2/p67phox), and a Rho GTPase (Rac1 or Rac2), and the membrane-associated catalytic core of NOX, including one of several NOX isoforms and the docking subunit p22phox, the latter of which is encoded by *CYBA* gene^14,15^. The catalytic subunits of NOXs have six or seven transmembrane domains with two heme-binding regions and a NADPH binding region to facilitate superoxide production^15^. The NOX-generated superoxide can be converted to other forms of ROS or detoxified by reduced glutathione and glutathione-coupled antioxidant enzymes, such as glutathione peroxidases (GPXs) and glutathione reductase (GR), to maintain redox homeostasis. Mounting evidence supports the notion that oxidative stress is a common feature of human cancers^16–18^.

In addition to the abnormally elevated NOX activity that accentuates oxidative stress, a marked elevation in antioxidant levels is another unique metabolic feature of oncogenic RAS activation^19^. Glutathione, a cellular thiol serving as a major determinant of cellular redox equilibrium, was found to be enriched through increased biosynthesis in cells with oncogenic KRAS^19^. This finding is consistent with studies showing that the activation of oncogenic RAS increased the total cellular glutathione pool^12,20,21^. Furthermore, stabilization of NRF2 in cancer cells harboring oncogenic RAS induces genes encoding stress-responsive enzymes, including GPXs, NAD(P)H:quinone oxidoreductase-1 (NQO1), glutamate-cysteine ligase (GCL), GR, heme oxygenase-1 (HO-1), and glutathione S-transferase (GST), some of which are directly involved in cellular glutathione biosynthesis and ROS detoxification^22–24^. Elevated antioxidant capacity underlies an important mechanism by which the oncogenic RAS-transformed cells adapt to adverse oxidative stress conditions while fueling rapid growth and proliferation^25,26^.

Cancer cells expressing oncogenic *RAS* appear to exhibit both an enhanced NOX activity, which increases ROS levels, and an increased cellular glutathione pool, which counteracts oxidative stress. We propose that these seemingly contrary events result in a higher level of redox balance, which serves to facilitate malignant transformation; on the other hand, this prominent metabolic feature would render cancer cells highly vulnerable to simultaneous inhibition of both pathways. Based on this hypothesis and our previous work^10^, we tested in this study the combinatory killing effects of BSO, a potent specific inhibitor of GCL in glutathione biosynthesis^27^, and DPI, a widely used NOX inhibitor^28^, on *HRAS*^*G12V*^-transformed ovarian epithelial cells, mutant *KRAS-*bearing pancreatic and colon cancer cells, and murine pancreatic cancer cells harboring both KRAS and p53 mutations. We observed a considerably elevated sensitivity of these diverse types of cancer cells to the combined BSO and DPI treatment in vitro, as well as a high efficacy of this treatment regime on suppressing the growth of cancer xenografts in athymic nude mice in vivo. Our study demonstrates that the combined DPI and BSO treatment exerts a high lethality to cancer cells harboring intractable RAS oncogenes and may hold great potential for clinical application.

## Materials and methods

### Genetically engineered mouse strains

*Kras*^*LSL-G12D/+*^ mice^29^ and *fElas*^*CreERT*^ mice^30,31^ were crossed to generate *fElas*^*CreERT*^*;Kras*^*LSL-G12D/+*^ double-transgenic mice (called *Kras*^*G12D/+*^ mice after Tamoxifen induction). *fElas*^*CreERT*^;*Trp53*^*LSL-R172H*/+^ mice^31^ were crossed with *fElas*^*CreERT*^*;Kras*^*LSL-G12D/+*^ mice to generate *fElas*^*CreERT*^*;Kras*^*LSL-G12D/+*^*;Trp53*^*LSL-R172H*/+^ mice (called KPC mice after Tamoxifen induction). At 60 days of age, male and female *fElas*^*CreERT*^, *KRAS*^*G12D/+*^, and KPC mice were randomly recruited and treated with tamoxifen (TM). Three weeks post TM induction, the pancreata were collected for experiments. All these animal experiments were reviewed and approved by the Stony Brook University Institutional Animal Care and Use Committee (IACUC).

### Cell lines and reagents

The immortalized non-tumorigenic normal ovarian epithelial T72 cell line and the tumorigenic *HRAS*^*G12V*^-transformed T72Ras cell line were generated as previously described^12,32^. Both T72 and T72Ras cells were cultured in a 1:1 mixture of M199 and MCDB105 (Sigma-Aldrich) with 10 ng/ml epidermal growth factor and 15% fetal bovine serum. Panc-1 cells were human patient-derived pancreatic ductal adenocarcinoma (PDAC) cancer cell line (from ATCC) that carries both oncogenic *KRAS* and *p53* mutations^33^. Murine KPC cells were generated from PDAC tumors of KPC mice carrying both *KRAS*^*G12D*^ and *p53*^*R172H*^ mutations^34^. L3.6pl cells, which were generated from metastatic liver nodules of a pancreatic cancer patient^35^ carrying the *KRAS*^*G12D*^ mutation^36^, were generously provided by Dr. Isaiah J. Fidler (The University of Texas MD Anderson Cancer Center, Houston, TX). Panc-1, L3.6pl, and KPC cells were cultured in DMEM (Gibco) supplemented with 10% fetal bovine serum (FBS), penicillin, and streptomycin (Invitrogen). HCT116 p53^+/+^ colon cancer cells (called HCT116^+/+^) carrying wild-type p53 and their isogenic HCT116 p53^−/−^ cells (called HCT116^−/−^) were cultured in McCoy5 (Hyclone) medium with 10% FBS. All the cells were free of mycoplasma contamination. DPI, BSO, N-acetyl-L-cysteine (NAC), and propidium iodide (PI) were purchased from Sigma, and Annexin V was from BD Biosciences. The different ranges of the effective concentrations of DPI and BSO for different cell lines or xenografts were pre-determined in pilot experiments or followed our previous studies.

### Assays for cellular total glutathione (GSX)

Cellular glutathione was measured using a glutathione assay kit (Beyotime Institute of Biotechnology, Shanghai, China) according to the manufacturer’s instruction. Briefly, L3.6pl, Panc-1, or KPC cells were treated with different concentrations of BSO in a six-well plate for 24 or 72 h. After treatment, cells were harvested and homogenized, and the cell content was deproteinized. Total cellular glutathione levels were determined by measuring the product of glutathionylated DTNB by a UV spectrophotometer at 412 nm. A standard curve was generated for calculating cellular glutathione levels.

### Determination of cell death

Cell death was determined by flow cytometry after cells were double-stained with annexin-V and PI (BD PharMingen, San Diego, CA). Briefly, after drug treatment, cells were harvested and washed twice with cold PBS and then stained with annexin-V for 15 min at room temperature in the dark, followed by staining with PI. The levels of cell death were then determined by measuring the fluorescence intensity of the cells using a BD FACSCalibur flow cytometer equipped with CellQuest Pro software. Ten thousand cells were analyzed per sample.

### Colony formation assay

HCT116^+/+^ and HCT116^−/−^ cells were seeded in six-well plates. Culture media were replaced with 3 ml of fresh media containing BSO and/or DPI at the specified experimental concentrations, and cells were incubated for 14 days. Survival colonies were stained with 0.2% crystal violet (Sigma).

### Gene expression analysis

The expression levels of glutamate-cysteine ligase catalytic subunit (*GCLC*), glutamate-cysteine ligase regulatory subunit (*GCLM*), glutathione synthetase (*GSS*), *GR, GPX1*, and *GPX4* genes relative to β-actin were analyzed by qRT-PCR as described^10^. Total RNA isolation, 1st strand cDNA synthesis, quantitative PCR were performed as described. Briefly, cell pellets or tissues were homogenized in 1 ml TRIzol Reagent (Ambion, Life Technologies, CA), total RNAs were separated from other cell contents by sequential chloroform and isopropanol precipitation. Aliquots of RNA samples were quantified and examined before reverse transcription using a Nanodrop spectrophotometer. The primer sets used for gene expression analysis by qRT-PCR include *CYBA*, forward 5′-GATCGAGTGGGCCATGT-3′ and reverse 5′-TGCTTGATGGTGCCTCC; *GCLC*, forward 5′-GGCACAAGGACGTTCTCAAGT-3′ and reverse 5′-CAAAGGGTAGGATGGTTTGGG-3′; *GCLM*, forward 5′-TGTGTGATGCCACCAGATTTG-3′ and reverse 5′-CGTGCGCTTGAATGTCAGG-3′; *GSS*, forward 5′-CTCTACGGCTCACCCAATGC-3′ and reverse 5′-TCGTCGGATCACATGGATGTT-3′; *GSR (GR)*, forward 5′-AGTGATCCCAAGCCCACAATA and reverse 5′-CACCAATGTAACCTGCACCAA; *GPX1*, forward 5′-CTCTTCGAGAAGTGCGAGGT-3′ and reverse 5′-GATGTCAGGCTCGATGTCAA-3′; and *GPX4*, forward 5′-AGTGAGGCAAGACCGAAGTAA-3′ and reverse 5′-TCCTGCTTCCCGAACTGGT-3′. Gene expression levels were determined by qRT-PCR in the Quantifast SYBR Green PCR mix (Qiagen GmbH, Germany) with a reaction condition of initial denaturation at 95 °C for 5 min and then 40 cycles of 95 °C for 10 s and 60 °C for 30 s in QuantStudio 3 machine. The comparative threshold Ct method was used with β-actin as an internal reference. Data quantification and statistical analysis were performed in Microsoft Excel and GraphPad Prism 6.0.

### Protein isolation and Western blot analysis

To evaluate protein levels, cell lysates were separated by SDS-PAGE and analyzed by Western blot as described^10^. Briefly, cell pellets or snap-frozen tissues were homogenized in a lysis buffer containing 10 mM Tris-HCl, pH 6.8–7.5, 2 mM EDTA, 0.5% SDS, and freshly added 2-mercaptoethanol. Tissue homogenates were centrifuged at 12,000 *g* for 15 min at 4 °C, and the supernatants were collected. The total protein extracts were aliquoted to determine protein concentration using a protein assay dye reagent concentrate (Bio-Rad, CA, USA). About 100 μg total protein was separated by SDS-PAGE and then transferred to nitrocellulose membrane. The membranes were probed with the following antibodies against p22phox (1:500; sc-20781, Santa Cruz), GCLC (1:600; sc-28965, Santa Cruz), GR (1:500; sc-13324, Santa Cruz), and β-actin (1:10000, Sigma-Aldrich), washed with PBST and then probed with their respective secondary antibodies conjugated to horseradish peroxidase for 1 h at room temperature. Autoradiography or the Odyssey Imaging System (LiCor Biosciences, Lincoln, NE) was used to visualize protein bands. Stripping buffer (Thermo, MA, USA) was used for sequential blotting and probing with other antibodies. ImageJ densitometry software was used to quantify individual bands.

### Xenograft mouse model

Isolated murine KPC cancer cells were cultured in DMEM medium containing 10% FBS to 90% confluence and collected in 1 × PBS. A total of 32 athymic nude mice at the age of 9 weeks were each injected with 5 × 10^5^ KPC cells subcutaneously. These mice were randomly divided into four groups. Nine days after injection, the mice were treated with PBS (control), BSO (500 mg/kg/day), DPI (6 mg/kg/day), or a mixed solution of 500 mg/kg BSO and 6 mg/kg DPI per day. Bodyweight and tumor size were measured after the day of KPC cell injection. All mice were sacrificed at day 24 (empirically determined) when an unbearable tumor burden (defined as a nodule of 18 mm diameter at any dimension) emerged in these mice. Tumor size was calculated based on the following equation: tumor volume (mm^3^) = *L* × *W* × [(*L* + *W*)/2] × 0.526, where *L* is the length and *W* the width^10^. The experimental treatment schemes for the mice were kept blind to the researchers when the mouse body and tumor parameters were analyzed. KPC cell xenograft mouse models were approved by the IACUC at Changhai Hospital.

### Statistics

In flow cytometry analysis, the Kolmogorov–Smirnov test (Cell Quest Pro software, Becton-Dickinson, San Jose, CA) was used for statistical evaluation among groups. All other statistical analyses were carried out using the Student’s unpaired *t* test (Prism GraphPad, San Diego, CA). The comparison of mouse body weight and tumor growth was carried out using two-way ANOVA. A *p*-value of <0.05 is considered to be statistically significant.

## Results

### Oncogenic HRAS^G12V^-transformed ovarian epithelial cells are vulnerable to the combined BSO and DPI treatment

**Fig. 1:**
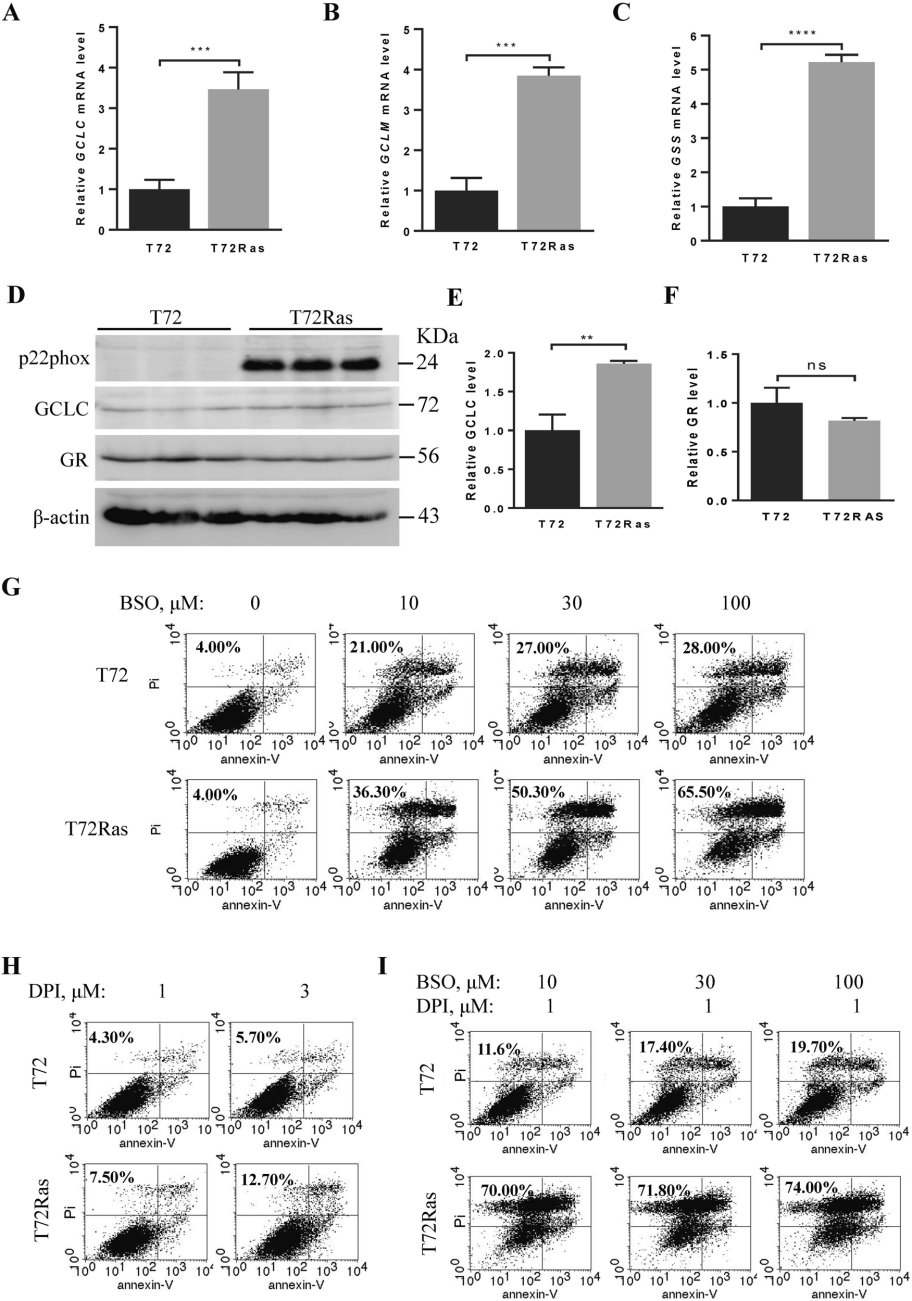
Selective cytotoxic effect of BSO and DPI combinatory treatment on oncogenic *HRAS*^*G12V*^-transformed ovarian epithelial cells. **A**–**C** qRT-PCR analysis of the expression levels of *GCLC*, *GCLM*, and *GSS* genes in T72 and T72Ras cells. β-actin serves as a control. **D** Western blot analysis of p22phox, GCLC, and GR protein levels in T72 and T72Ras cells. β-actin serves as a loading control. **E**–**F** Quantitative analysis of GCLC and GR protein levels in **D**. **G** T72 parental cells and their isogenic *HRAS*^*G12V*^-transformed T72Ras cancer cells were treated with glutathione biosynthesis inhibitor BSO at 0, 10, 30, or 100 μM for 30 h. Cell viability was measured by flow cytometry with annexin-V/PI double staining. The numbers shown at the upper left of each panel indicate the percentage of the annexin-V/PI positive cells. **H** T72 and T72Ras cells were treated with DPI at 1 or 3 μM for 30 h. Cell viability was measured as described in **G**, and cell death was expressed as the percentage of the annexin-V/PI positive cells. **I** Combined treatment using BSO (10, 30, or 100 μM) and DPI (1 μM) for 30 h induced significant death of T72Ras cells (lower panel) but not parental T72 cells (upper panel). Data are mean ± SD of three independent experiments with Student’s *t* test. **p < 0.01, ***p < 0.001, ****p < 0.0001. KDa kilodalton.

Our previous studies showed that oncogenic HRAS^G12V^ transformation of T72Ras cells led to increases in the expression of genes encoding NOX components, NOX activity, and ROS compared to their parental ovarian epithelial T72 cells^10,12^. Studies also showed a compensatory increase in cellular glutathione in T72Ras cells that exhibit malignant behavior for generating xenograft tumors as compared to T72 cells^10,12,32^. To determine the antioxidant capacity of T72Ras cells, we analyzed the expression of *GCLC, GCLM*, and *GSS* in the glutathione synthesis pathway, as well as glutathione-associated H_2_O_2_ detoxification genes, including *GR, GPX1*, and *GPX4*. Among these genes, the expression levels of *GCLC, GCLM*, and *GSS* were significantly upregulated in T72Ras cells compared to those in T72 cells (Fig. 1a–c), which is in line with previous findings that T72Ras cells have a higher level of cellular glutathione than T72 cells^12^. Western blot analysis revealed that the levels of p22phox were prominent in T72Ras cells (Fig. 1d–f). Although no significant alterations were observed in GR protein levels, GCLC protein level increased significantly in T72Ras cells compared to that in T72 cells (Fig. 1d–f). These data suggest a simultaneous elevation in both the ROS-generating NOX and the ROS-scavenging glutathione biosynthesis upon the transformation of T72 cells by oncogenic HRAS^G12V^.

To determine whether these metabolic alterations create vulnerability in cancer cells to the concurrent inhibition of the elevated NOX activity and glutathione biosynthesis, we first evaluated the cytotoxic effects of inhibiting glutathione biosynthesis comparatively on these isogenic cell lines. As illustrated in Fig. 1g, reduction of total cellular glutathione by BSO treatment alone at 10, 30, or 100 μM for 30 h in T72 cells induced cell death ranging from 21.00 to 28.00% as probed by annexin-V/PI in flow cytometric analysis. In contrast, T72Ras cells were more sensitive to BSO treatment than T72 cells, with significantly increased cell death ranging from 36.30 to 65.50%, indicating an increased sensitivity of T72Ras cells to the inhibition of glutathione biosynthesis. We then evaluated the cytotoxic effects of NOX inhibitor DPI. T72 cells exhibited a basal level of sensitivity to DPI at 1 and 3 μM with cell death ranging from 4.30 to 5.70%, while T72Ras cells had a little increase in cell death ranging from 7.50 to 12.70% after being treated for 30 h (Fig. 1h). In marked contrast, a combined treatment with 1 μM DPI and 10 μM BSO significantly enhanced cytotoxicity to T72Ras cells with 70.00% cell death vs. 11.60% in T72 cells (Fig. 1i). This combined regimen resulted in approximately a 6-fold increase in the death of T72Ras cells over T72 cells, a more than 9-fold increase over T72Ras cells with 1 μM DPI treatment alone, and about a 2-fold increase with 10 μM BSO treatment alone (Fig. 1g–i). Increasing BSO concentrations to 30 μM and 100 μM did not significantly increase the cytotoxic effect on T72Ras cells, which exhibited a rate of cell death ranging from 71.80 to 74.00%, compared to the slightly increased killing effect on T72 cells ranging from 17.40 to 19.70%. Overall, these data suggest that the *HRAS*^*G12V*^-transformed cells are highly sensitive and selective to the combinatory therapy of BSO and DPI compared to the non-transformed isogenic cells.

### Pancreatic cancer patient-derived L3.6pl cells bearing oncogenic KRAS are vulnerable to BSO and DPI combination therapy

**Fig. 2:**
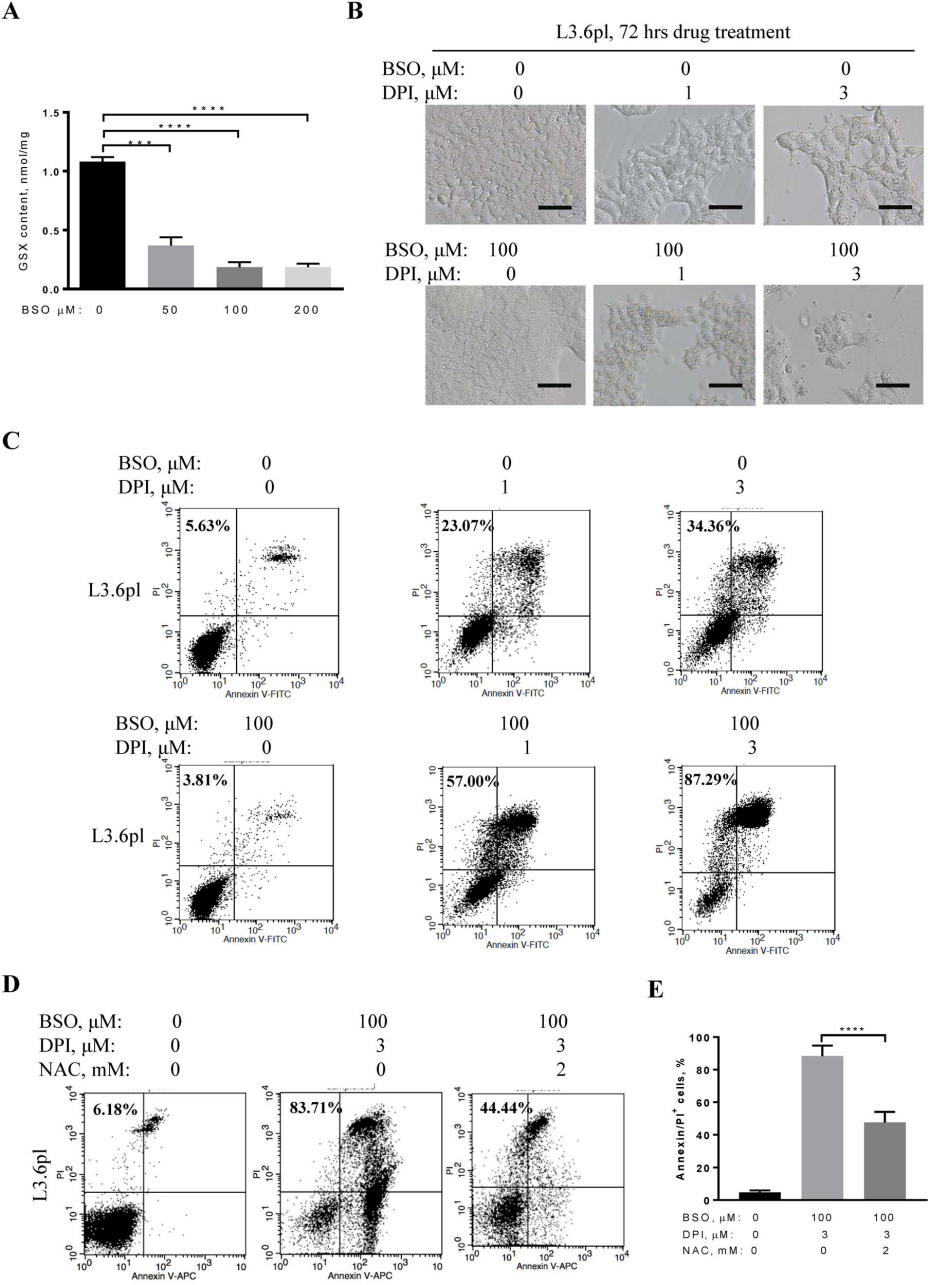
Cytotoxicity induced by BSO and DPI combinatory treatment in highly metastatic pancreatic cancer cells. **A** Total cellular glutathione (GSX), including reduced and oxidized glutathione, was measured after 72 h of BSO treatment at 0, 50, or 100 μM in L3.6pl cells by spectrophotometry. **B** L3.6pl cells were treated with DPI alone (1 or 3 μM), BSO (100 μM) alone, or BSO (100 μM) and DPI (1 or 3 μM) for 72 h. Cell morphology and population growth were compared under a light microscope. Scale bar, 200 μm. **C** L3.6pl cells were treated as described in **B** for 72 h. The cell death rate was measured by flow cytometry using annexin-V and PI as probes and expressed as a percentage of the annexin-V/PI positive cells. L3.6pl cells without any treatment served as controls. **D** L3.6pl cells were treated with a combination of 3 μM DPI and 100 μM BSO in the presence or absence of 2 mM NAC for 72 h. Cell death rates were determined by Annexin V/PI double staining. **E** Statistic analysis of cell death rate of L3.6pl cells treated in **D**. Data are mean ± SD of three independent experiments with Student’s *t* test. ***p < 0.001, ****p < 0.0001.

The selectivity and sensitivity of *HRAS*^*G12V*^-transformed cells to the combined BSO and DPI treatment motivated us to test if patient-derived cancer cells carrying oncogenic RAS are also sensitive to this therapy. Oncogenic *KRAS*^*G12D*^*-*bearing L3.6pl cells are highly aggressive pancreatic cancer cells derived from a patient of PDAC with liver metastasis^36^. As the concentration of BSO used for effective inhibition of cellular glutathione biosynthesis varies with different cell types^19,26^, we first measured cellular total glutathione levels of L3.6pl cells after treatment with BSO at concentrations of 0, 50, 100, or 200 μM for 72 h. As shown in Fig. 2a, 50 μM BSO treatment resulted in a 65.82% reduction of total cellular glutathione (*p* < 0.001, *n* = 3) compared to the control group, while 100 and 200 μM BSO treatments reduced total cellular glutathione by 82.98% and 82.87%, respectively. Therefore, we chose a BSO concentration of 100 μM, the lowest concentration that induces a maximum glutathione reduction. 100 μM BSO treatment alone for 72 h induced no significant cytotoxicity in L3.6pl cells, while DPI treatment at 1 and 3 μM decreased cell viability compared to without any treatment. When combined with 100 μM BSO, viable cells drastically decreased in a DPI dose-dependent manner (Fig. 2b). Evaluation of cell death with annexin-V/PI staining revealed that 1 and 3 μM of DPI treatment for 72 h led to a dose-dependent increase in cell death at 23.07% and 34.36%, respectively (Fig. 2c). BSO at 100 μM exerted no significant cytotoxicity to L3.6pl cells, even though a reduction of about 83% in glutathione was observed (Fig. 2a, c). Strikingly, combination treatment with BSO and DPI was effective in inducing L3.6pl cell death, which increased to 57.00% under 100 μM BSO and 1 μM DPI and to 87.29% under 100 μM BSO and 3 μM DPI for 72 h (Fig. 2c). These results indicate a cooperative cytotoxic effect of combined treatment on L3.6pl cells.

To evaluate whether this remarkably cooperative killing effect is dependent on a redox-mediated mechanism, we used NAC, an N-acetyl derivative of L-cysteine and a precursor in the formation of glutathione, to replenish the reduced cellular glutathione pool. After incubation with 100 μM BSO and 3 μM DPI in the presence of 2 mM NAC for 72 h, the death rate of L3.6pl cells significantly decreased from 88.38 ± 6.46% in the absence of NAC to 47.51 ± 6.57% in the presence of NAC (Fig. 2d, e). These results suggest that the cooperative killing effect induced by BSO and DPI combination treatment involves, at least in part, a redox-mediated mechanism, which also reflects the acquired dependence of cancer cells on elevated glutathione.

### Colon cancer cells harboring oncogenic KRAS are sensitive to BSO and DPI combined therapy

**Fig. 3:**
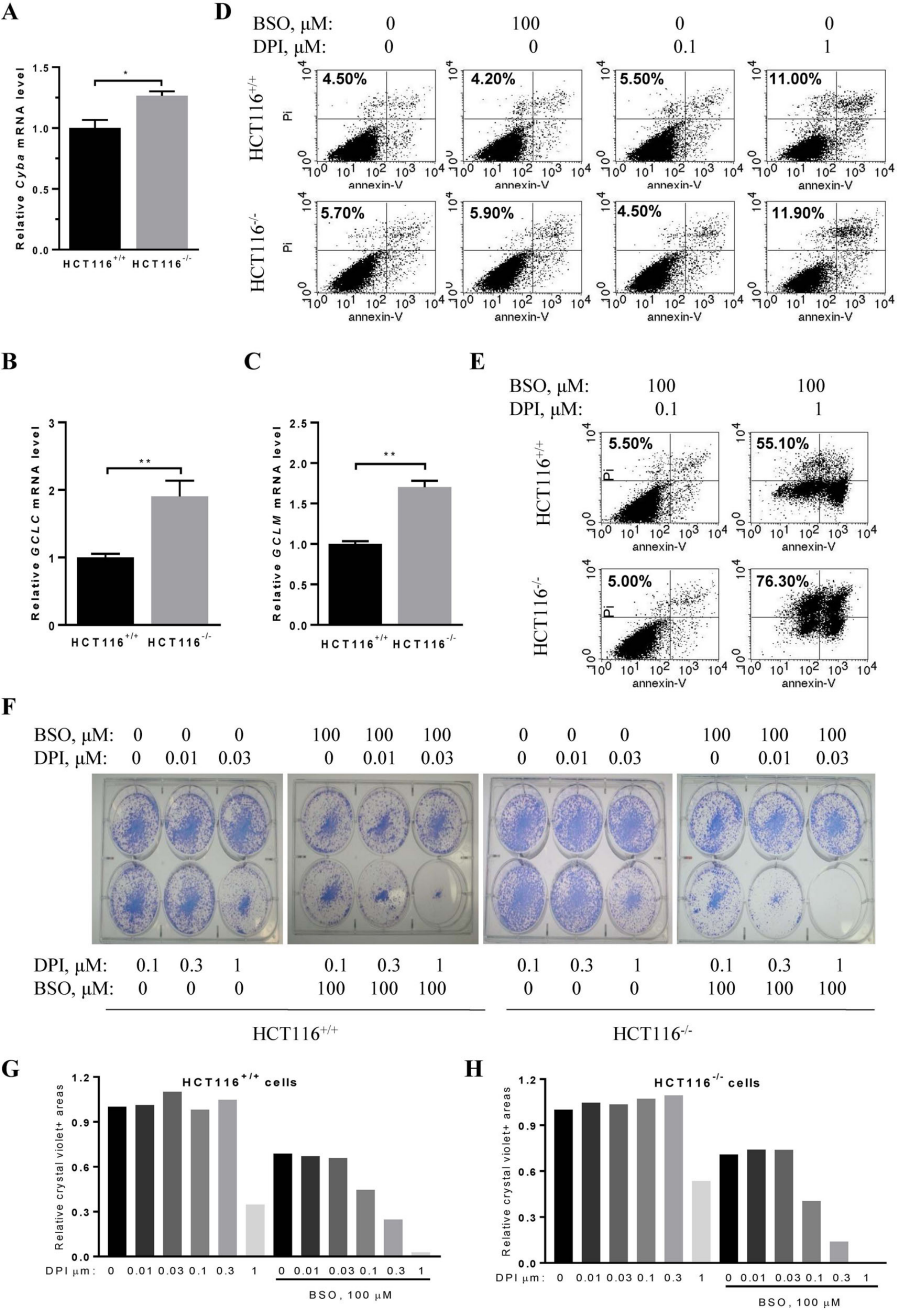
BSO and DPI cooperatively induced death of oncogenic *KRAS*-expressing colon cancer cells with or without p53 mutation. **A**–**C** qRT-PCR analysis of the expression levels of *CYBA* (the gene encoding p22phox), *GCLC*, and *GCLM* genes in HCT116^+/+^ and HCT116^−/−^ cells. β-actin serves as a control. **D** HCT116^+/+^ and HCT116^−/−^ cells were incubated with BSO (100 μM) alone or DPI alone (0.1 μM or 1 μM) for 24 h and cell death was measured by flow cytometry using annexin-V and PI as probes. **E** HCT116^+/+^ and HCT116^−/−^ cells were incubated with 0.1 μM or 1 μM DPI in the presence of 100 μM BSO for 24 h. The cell death rate was determined with flow cytometry. **F** Equal numbers of HCT116^+/+^ and HCT116^−/−^ cells were seeded in 6-well plates and treated with DPI at 0, 0.01, 0.03, 0.1, 0.3, or 1 μM in the presence or absence of 100 μM BSO for 14 days. Cells were stained with crystal violet and photographed. **G**, **H** Bar graph presentation of data analysis shown in **F**. The density of the crystal violet areas in the colonies of either cell line was considered as 1 (100%) for the respective data set. Data are mean ± SD of three independent experiments with Student’s *t* test. *p < 0.05, **p < 0.01.

p53 mutations significantly promote invasive cancer formation in the presence of RAS mutations, which are often observed in many cancer patients^34^. Our previous studies have shown that KRAS-mutated colon cancer cells with p53 deletion have a higher NOX activity and are sensitive to NOX inhibition compared to cells with wild-type p53^10^. Here, by using qRT-PCR analysis on a pair of isogenic colon cancer cell lines, the HCT116 cells harboring mutant *KRAS* with wild-type *p53* (called HCT116^+/+^) or *p53* deletion (HCT116^−/−^), we found that the expression levels of *CYBA, GCLC*, and *GCLM* significantly increased in HCT116^−/−^ cells compared to that in HCT116^+/+^ cells^8,10^ (Fig. 3a–c).

The simultaneous elevation of both ROS-generating NOX and ROS-scavenging GCL in HCT116^−/−^ cells inspired us to test the effectiveness of BSO and DPI combination therapy. Treatment with 100 μM BSO or 0.1 μM DPI alone for 24 h did not induce significant cell death in either HCT116^+/+^ or HCT116^−/−^ cells compared to cells without any treatment (Fig. 3d). While treatment with DPI alone at 1 µM for 24 h only induced death in 11% of HCT116^+/+^ cells and 11.9% of HCT116^−/−^ cells (Fig. 3d), the combination with 100 μM BSO induced dramatic cell death in 55.10% of HCT116^+/+^ cells and 76.30% of HCT116^−/−^ cells (Fig. 3e), demonstrating the effectiveness of the combination treatment in killing cancer cells bearing oncogenic KRAS and p53 mutations. Given our previous studies showing that 10 µM DPI treatment for 24 h led to 17% cell death in HCT116^+/+^ cells and 32% in HCT116^−/−^ cells^10^, our data indicate that a 10-fold less concentration of DPI can reach a better efficacy when combined with BSO. Notably, these data also showed that HCT116^−/−^ cells are more sensitive to the combined treatment than HCT116^+/+^ cells (Fig. 3e). Colony formation assay confirmed that the combined treatment of BSO (100 µM) and DPI (>0.1 µM) substantially inhibited the colony formation ability of both HCT116^+/+^ and HCT116^−/−^ cells compared to any single treatment (Fig. 3f–h).

### BSO and DPI combined therapy effectively kills pancreatic cancer cells harboring both KRAS and p53 mutations

**Fig. 4:**
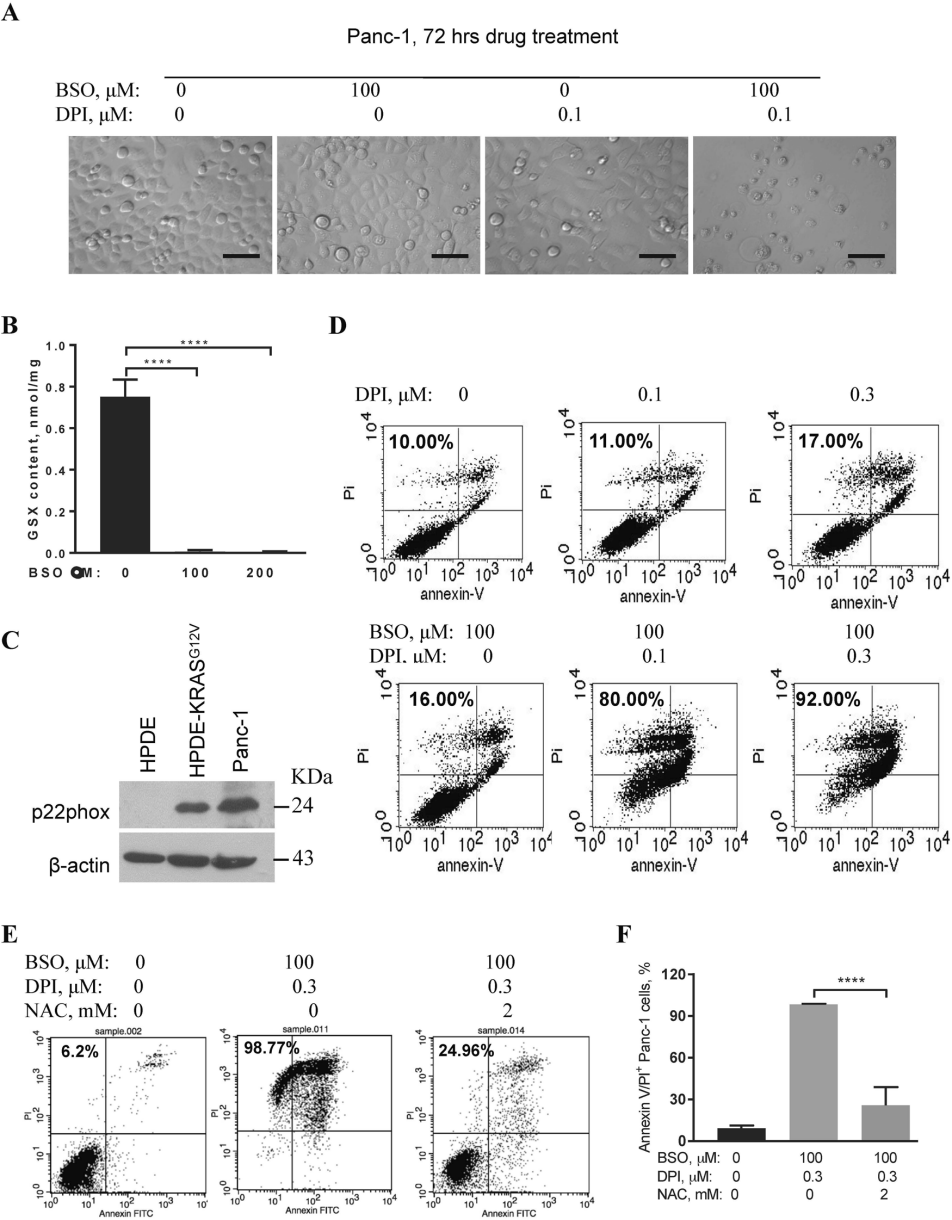
BSO and DPI cooperatively induce significant death of primary human pancreatic cancer cells. **A** Comparison of cell morphology and growth in Panc-1 cells after treatment with 0.1 μM DPI, 100 μM BSO, or the combined 100 μM BSO and 0.1 μM DPI for 72 h. DPI or BSO treatment alone had a limited impact on Panc-1 cells, while the combined treatment considerably reduced the number of living cells as observed under a light microscope. Scale bar, 200 μm. **B** Total cellular glutathione (GSX) levels were measured by spectrophotometry after 24 h of BSO treatment at 0, 100, or 200 μM in Panc-1 cells. Treatment was performed with three independent experiments. **C** Western blot analysis of p22phox protein levels in Panc-1 cells and oncogenic *KRAS*^*G12V*^-transformed HPDE cells as compared to parental HPDE cells. KDa, kilodalton. **D** Concurrent DPI and BSO treatment induced significant Panc-1 cell death. Cells were incubated with 0.1 or 0.3 μM DPI, 100 μM BSO, or their combination as indicated for 72 h, and cell death was determined by Annexin V/PI double staining. Panc-1 cells without any treatment served as the control. **E** Panc-1 cells were treated with 0.3 μM DPI and 100 μM BSO combination in the presence or absence of 2 mM NAC for 72 h. Cell death rates were determined by Annexin V/PI double staining. **F** Statistical analysis of the percent death rates of Panc-1 cells treated in **E**. Data are mean ± SD of three independent experiments with Student’s *t* test. ****p < 0.0001.

We further tested the cytotoxic effect of this combinatory treatment in PDAC cells harboring both *KRAS* and *p53* mutations. Panc-1 cells are derived from a poorly differentiated primary pancreatic cancer patient with both *KRAS* and *p53* mutations^33^. 100 μM BSO or 0.1 μM DPI treatment alone had no significant impact on Panc-1 cells, while joint treatment with BSO and DPI for 72 h effectively induced Panc-1 cell death (Fig. 4a). Since 100 μM BSO treatment did not induce any morphological changes to Panc-1 cells, we thought that BSO at this concentration might be insufficient in depleting cellular glutathione. However, measurement of total cellular glutathione content revealed that 100 μM BSO treatment for 24 h was sufficient to decrease the content to a nearly undetectable level as compared to the non-treatment control cells (Fig. 4b, *p* < 0.0001, *n* = 3). Western blot analysis revealed the prominent expression of p22phox in Panc-1 cells, as well as in KARS^G12V^-transfected normal human pancreatic ductal epithelial (HPDE-KRAS^G12V^) cells, compared to normal HPDE cells (Fig. 4c). Flow cytometry of Panc-1 cells with annexin V/PI staining showed that treatment with DPI alone at 0.1 and 0.3 μM for 72 h induced 11.00% and 17.00% of cell death, respectively (Fig. 4d, upper panel), and similarly, BSO treatment alone at 100 μM induced 16.00% of cell death (Fig. 4d, lower panel). In marked contrast, DPI treatment at 0.1 or 0.3 μM in combination with 100 μM BSO for 72 h induced 80.00% and 91.00% of death of Panc-1 cells, respectively (Fig. 4d, lower panel). These data indicate a high sensitivity of Panc-1 cells to the concomitant reduction in glutathione and NOX activity.

Antioxidant NAC was used to test if the cytotoxicity of the combined BSO and DPI treatment was mediated by a redox-related mechanism. Upon treatment with 2 mM NAC, cell death induced by the combination of 100 μM BSO and 0.3 μM DPI dropped from 98.34 ± 0.44% to 25.79 ± 12.99%, suggesting that an exogenous antioxidant supplement could largely attenuate the killing effect of the BSO and DPI combination (Fig. 4e, f). Thus, these results suggest that pancreatic cancer cells carrying both *KRAS* and *p53* mutations are vulnerable to BSO and DPI combinatory inhibition, and the cytotoxic effect of BSO and DPI was mainly triggered by a redox-mediated mechanism.

### BSO and DPI combined therapy effectively kills pancreatic cancer cells derived from murine KPC tumor

**Fig. 5:**
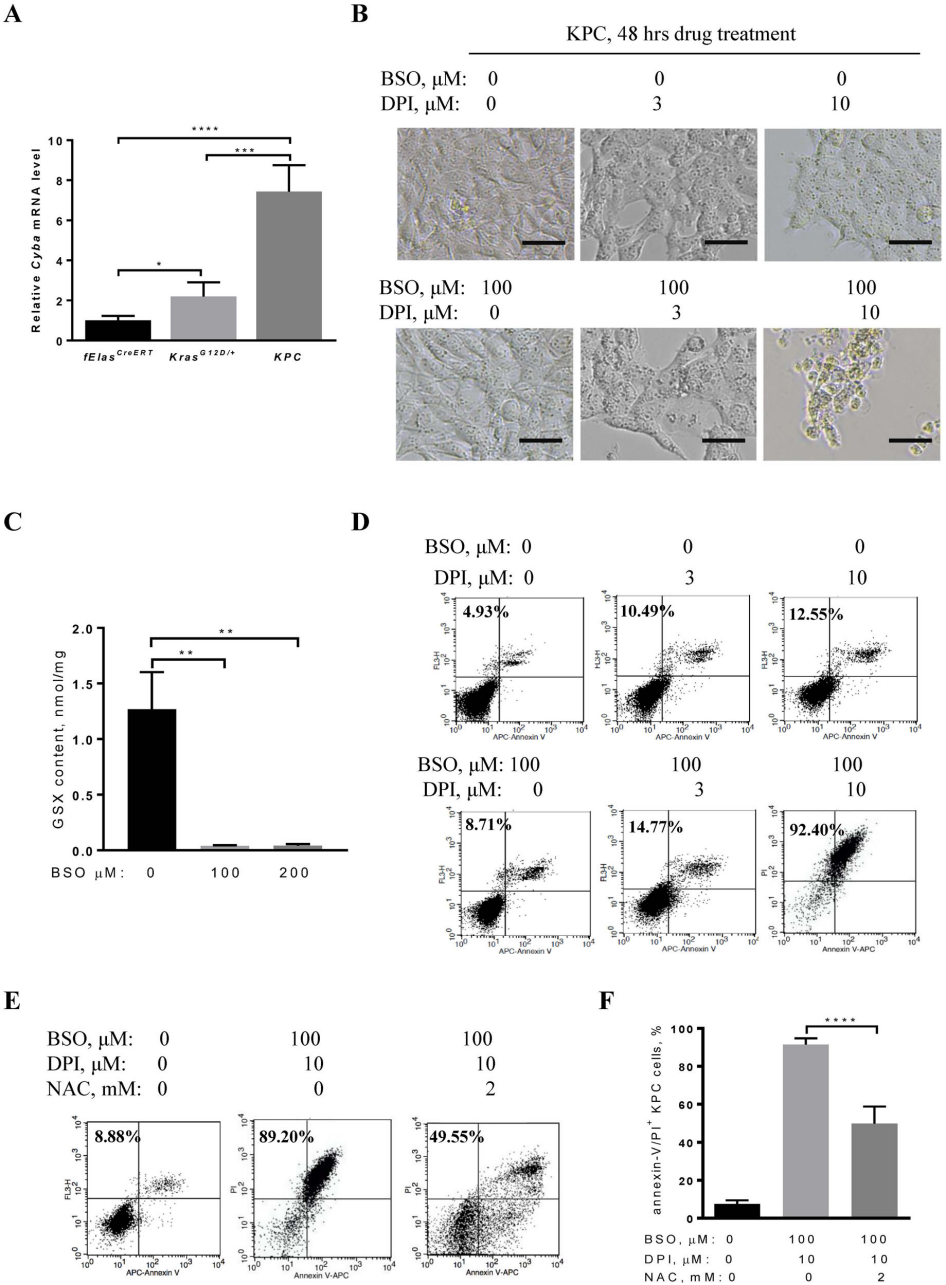
Cytotoxicity of BSO and DPI combinatory treatment in murine KPC cells. **A** qRT-PCR analysis of pancreatic *CYBA* gene expression. At 60 days of age, randomly recruited male and female *fElas*^*CreERT*^ (*n* = 4), *KRAS*^*G12D/+*^ (*n* = 3), and KPC mice (*n* = 6) were treated with TM. Three weeks post TM induction, the pancreata were collected for RNA extraction. β-actin serves as a control. **B** An equal number of KPC cancer cells were seeded in 6-well plates and treated with DPI at 0, 3, or 10 μM in the presence or absence of 100 μM BSO for 48 h. Cell morphology, population growth, and death were observed under a light microscope. KPC cells without any treatment served as the control. Scale bar, 200 μm. **C** Total cellular glutathione (GSX) was measured after 24 h of BSO treatment at 0, 100, or 200 µM in KPC cells by spectrophotometry. **D** KPC cancer cells were treated with 3 or 10 μM DPI alone, 100 µM BSO alone, or the combined DPI (1 or 3 μM) and BSO (100 μM) for 48 h. Cell death rates were probed by annexin-V/PI assay. KPC cells without any treatment served as the control. **E** KPC cells were treated with 10 μM DPI and 100 μM BSO in the presence or absence of 2 mM NAC for 48 h to assess the changes in cell death rates by flow cytometry using the annexin-V/PI assay. **F** Statistical analysis of KPC cell death rates in **E**. Data are mean ± SD of three independent experiments with Student’s *t* test. *p < 0.05, **p < 0.01, ***p < 0.001, ****p < 0.0001.

Studies have shown that mice expressing both *Kras*^*G12D/+*^ and *p53*^*R172H/+*^ at endogenous levels in the pancreata (called KPC mice) rapidly developed PDAC compared to mice expressing *Kras*^*G12D/+*^ alone^29,34,37^. To determine if the expression of both *Kras*^*G12D/+*^ and *p53*^*R172H/+*^ results in a higher level of *Cyba* expression, 60-day-old male and female mice carrying *Kras*^*G12D/+*^, *KPC*, and *fElas*^*CreERT*^ (control) were induced by tamoxifen. Our data revealed that three weeks after tamoxifen induction, pancreatic *Cyba* mRNA levels of KPC mice were significantly elevated, which were about 3.4 times and 7.4 times higher than that of the *Kras*^*G12D/+*^ mice and *fElas*^*CreERT*^ mice, respectively (Fig. 5a).

We then tested the combined killing effects on KPC mice-derived cancer cells. 100 μM BSO treatment alone did not cause any detectable change in the growth of KPC cells compared to cells without any treatment (Fig. 5b). Treatment with DPI alone at 3 and 10 μM for 48 h inhibited cell growth (Fig. 5b). However, upon treatment with a combination of 100 μM BSO and 10 μM DPI, KPC cell growth was further remarkably inhibited (Fig. 5b, lower panel). Glutathione assay revealed that 100 μM BSO treatment reduced glutathione levels by 96.90% (*p* < 0.01, *n* = 3) (Fig. 5c), even though only minimal cell death was observed (Fig. 5b). Flow cytometric analysis revealed that 100 μM BSO and 10 μM DPI combination induced cell death in 92.40% of KPC cells, which is in marked contrast to 8.71% of cell death by 100 μM BSO treatment or 12.55% of cell death by 10 μM DPI treatment (Fig. 5d). NAC treatment at 2 mM partially reversed the cytotoxicity of BSO and DPI combined treatment to KPC cells (Fig. 5e, f).

### BSO and DPI combined treatment inhibited pancreatic tumor growth in vivo

**Fig. 6:**
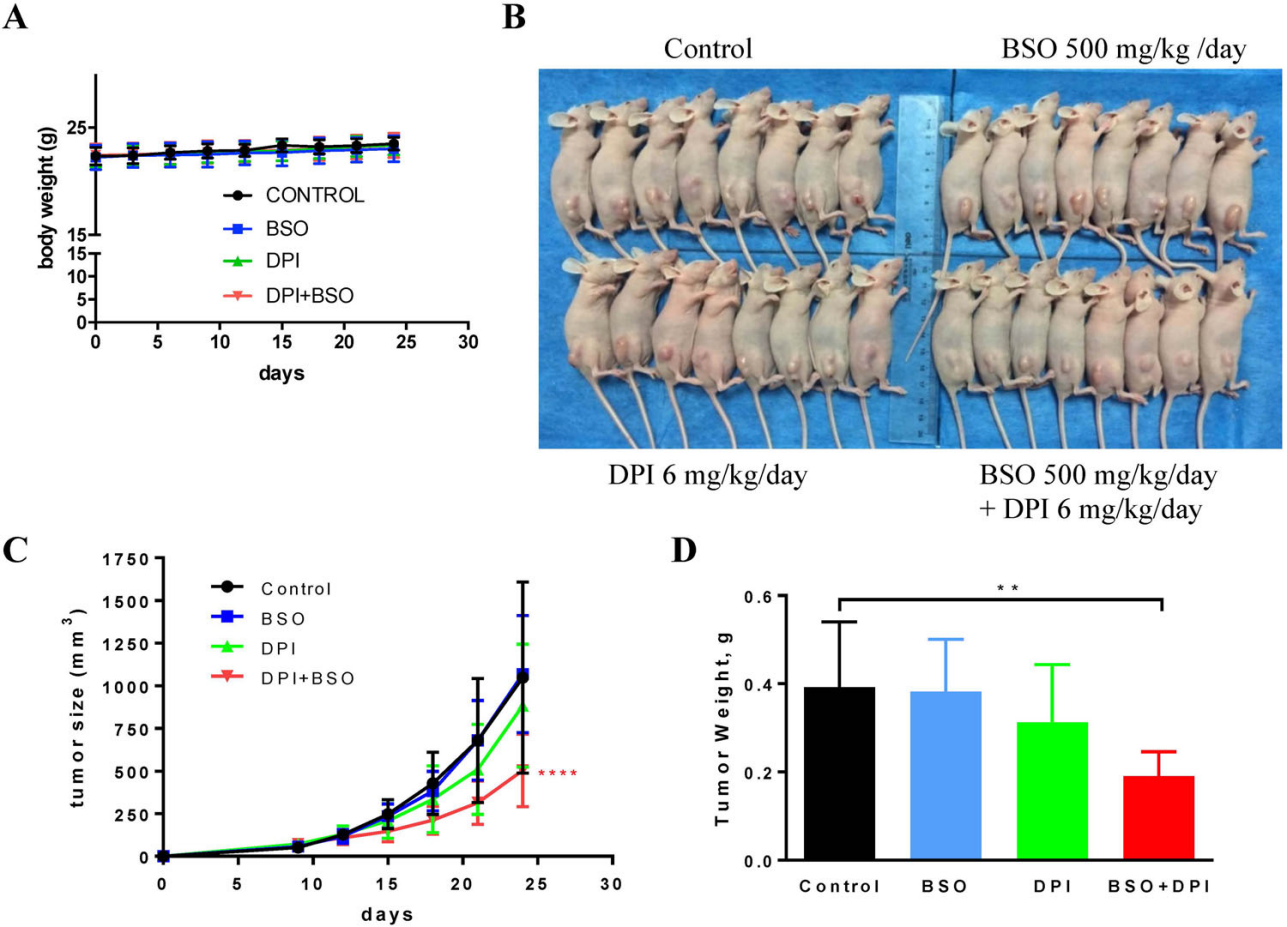
Inhibition of pancreatic tumor growth in vivo by BSO and DPI combinatory treatment. **A**–**D** Athymic nude mice (*n* = 32) were injected subcutaneously with 5 × 10^5^ KPC cells on the right flank at the age of 9 weeks and randomly divided into four groups (*n* = 8 per group). Treatment started 9 days after KPC cell injection with PBS, 500 mg/kg BSO, 6 mg/kg DPI, or 500 mg/kg BSO plus 6 mg/kg DPI. When the tumor diameter reached 18 mm in a few mice, all mice were sacrificed as mandated by the animal care protocol, and tumor nodules were excised for subsequent statistic comparison in size and weight. **A** Body weight changes in all 32 mice starting from KPC cell injection. No difference was noted in all four treatment groups. Two-way ANOVA was used for statistical analysis. **B** Photographic gross comparison of athymic nude mice bearing KPC cell xenografts on the right flank. **C** Tumor volumes were measured throughout the experiment to evaluate the effect of drug treatment using the following equation: tumor volume (mm^3^) = *L* × *W* × (*L* + *W*)/2 × 0.526. Data presented are mean tumor volume in mm^3^ ± SD. Two-way ANOVA was used for statistical analysis. ****p < 0.0001. **D** Statistic analysis of tumor weight among four treatment groups. Data presented are mean tumor weight ± SD (gram). Student’s *t* test. **p < 0.01.

The effectiveness of BSO and DPI combined therapy in killing *RAS*-bearing cancer cells in vitro motivated us to evaluate their tumor-suppressive effects in vivo. Athymic nude mice were inoculated subcutaneously with 5 × 10^5^ KPC cells, which were shown to be the least sensitive among all the tested cancer cells. The mice were then randomly divided into four groups and treated with PBS, 500 mg/kg BSO, 6 mg/kg DPI, or a combination of 500 mg/kg BSO and 6 mg/kg DPI. The body weight of the mice under any treatment regimen did not change significantly (Fig. 6a), suggesting that no noticeable side-effects were induced under these treatment conditions. Tumors in the PBS control group grew rapidly (Fig. 6b, c). Treatment with 500 mg/kg BSO caused no detectable changes in the size of xenograft tumors, and treatment with 6 mg/kg DPI alone caused a decrease of 18.5% in tumor size. By contrast, the group with BSO and DPI treatment showed a pronounced restriction on tumor size, with a 53.5% reduction after 24 days of treatment (Fig. 6b, c). Consistently, simultaneous inhibition of NOX activity and glutathione synthesis caused a notable reduction of 50.6% in tumor weight compared to no change with BSO treatment or 17.7% reduction with DPI treatment alone (Fig. 6d). These data indicate that the combined administration of BSO and DPI exerts a significant cooperative effect on deterring tumor growth in mice over any individual treatment regimen.

## Discussion

In this study, we have presented several lines of evidence to support a novel therapeutic strategy against oncogenic *RAS*-expressing cancer cells by concurrently targeting glutathione biosynthesis and NOX activity. We tested this strategy in oncogenic *RAS*-transformed cells, patient-derived cancer cells, and tumor xenografts in vivo. Our studies reveal that the combined DPI and BSO treatment (1) preferably killed the *HRAS*^*G12V*^-transformed human ovarian epithelial cells over their normal counterpart; (2) induced massive cell death in the highly metastatic human L3.6pl PDAC cells compared to the individual treatment; (3) exerted a potent and cooperative killing effect on oncogenic *KRAS-*expressing and p53-mutated human colon cancer cells; (4) induced significant cell death of both human and murine PDAC cells harboring *KRAS* and *p53* mutations; (5) suppressed tumor growth of KPC cell xenografts in vivo compared to the individual treatment regimens. Furthermore, the supplement of NAC, a glutathione precursor disrupting the cooperation of BSO and DPI, significantly attenuated the cytotoxic effects of the combination therapy. These results suggest an acquired vulnerability of oncogenic *RAS*-bearing cancer cells to the concurrent inhibition of NOX and glutathione biosynthesis. Such a vulnerability caused by oncogenic RAS is in line with evidence showing that cancer cells with oxidative stress are increasingly dependent on the enhanced antioxidant for better survival and aberrant proliferation in adverse metabolic conditions^12^. Antioxidant signature marked by the elevated glutathione synthesis has been recognized as an important metabolic feature of oncogenic *KRAS*-mediated malignant transformation^19^. Glutathione pool at an elevated level serves to detoxify the damaging effects of increasing ROS, and therefore, is crucial for the adaptation of cancer cells to oxidative stress for optimal survival and growth^16,17^. Accordingly, inhibition of glutathione biosynthesis has been harnessed as a therapeutic strategy in various preclinical models, including KRAS-driven pancreatic cancer and non-small cell lung cancer^19,20,38^. However, clinical efficacy has always been a limiting issue. Our results have identified DPI as a partner of BSO in exerting profound cytotoxic effects on cancer cells bearing oncogenic *RAS*.

**Fig. 7:**
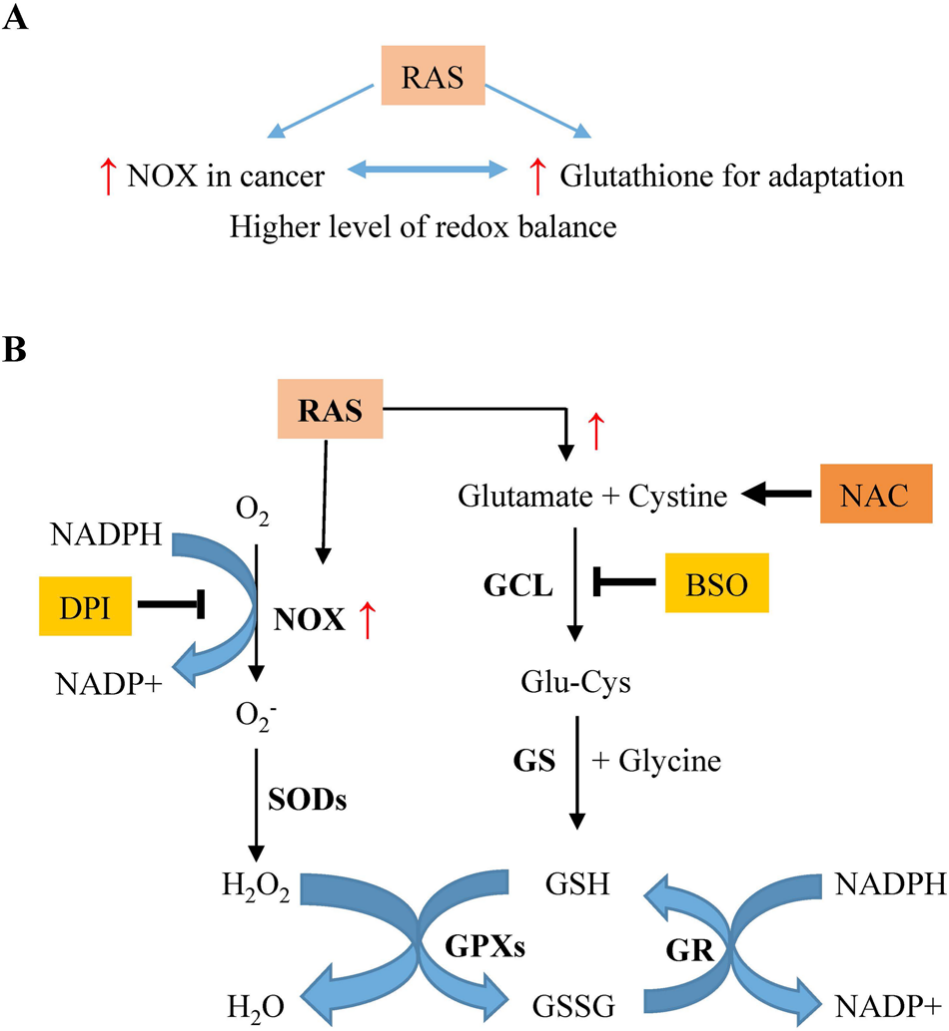
Targeted inhibition of NOX activation and glutathione biosynthesis for treating mutant *RAS*-harboring cancers. **A** Oncogenic RAS activation enhances both ROS-generating NOX and ROS-scavenging glutathione biosynthesis to induce a higher level of redox balance for optimal survival and proliferation of cancer cells. **B** NOX catalyzes the oxidation of NADPH to NADP^+^, leading to the generation of O_2_^−^ by the reduction of molecule oxygen (O_2_). O_2_^−^ can be converted to other forms of reactive oxygen species (ROS), the levels of which can be regulated by the reduced glutathione and glutathione-coupled antioxidant enzymes. Normal cells keep the ROS and antioxidant capacity at basal levels. However, oncogenic RAS activates NOX activity, leading to an increase in ROS generation. It also upregulates GCL and other enzymes in glutathione biosynthesis, leading to an elevated cellular glutathione pool. As a result, the heightened oxidative stress and antioxidant capability, at a certain threshold with an adequate range, achieve a higher level of redox balance, on which cancer cells depend to gain a selective advantage on survival and proliferation in adverse conditions. On the other hand, the coexistence of both elevated redox arms irrevocably creates a vulnerability and, thus, an opportunity for the targeted therapy, as exemplified by the combined DPI and BSO treatment in this study. Exogenous NAC can, directly and indirectly, replenish the reduced cellular glutathione pool to significantly attenuate the cytotoxic effects of the combination therapy.

Targeted inhibition of ROS-generating NOX and ROS-scavenging glutathione biosynthesis seems contradictory; however, activation of NOX and enhancement of glutathione biosynthesis have been consistently observed to coexist in *RAS*-mutated cancers^10,12^. These two events cooperate to achieve a higher level of redox balance (Fig. 7a, b) compared to that in the normal cell counterparts, and thus, endow cancer cells with a selective advantage on proliferation with optimal survival over normal cells under adverse conditions^16,17^. Furthermore, the redox system not only involves redox-associated enzymes, such as the glutathione biosynthesis pathway, GPXs, GR, catalase, superoxide dismutase (SOD), and NOXs, but also intertwines with glucose metabolism, fatty acid oxidation, and mitochondrial function for the generation of reducing equivalent NADPH, with glutamine metabolism for the supplement of glutamate for glutathione synthesis, and with other signaling pathways including NRF2-mediated upregulation of the antioxidant system for a better adaptation to oxidative stress. Thus, the functions of the redox system are multifaceted, which underscore the necessary co-existence of NOX activation and enhancement of glutathione pool acquired as two important survival and proliferation features in cancers. By contrast, normal cells appear to maintain only a basal homeostatic level of antioxidant capacity with minimal oxidative stress, and therefore, are less sensitive to the inhibition of these two redox arms.

Overall, our results have shown that the acquired dual elevations in NOX activity and glutathione pool create a therapeutic vulnerability in oncogenic RAS-transformed cancer cells. Targeting such a unique vulnerability by concurrently inhibiting NOX and glutathione synthesis induces a cooperative lethality highly potent to cancer cells harboring oncogenic RAS. Thus, our results not only have potentially important mechanistic implications in how both the oxidative and antioxidant arms of redox homeostasis cooperate to regulate cell survival and death, but also provide a promising therapeutic strategy for cancer patients harboring oncogenic RAS that warrants further translational and clinical investigation.
